# The Declaration on Palliative Care in a Pandemic: report of the African Ministers of Health Meeting and the 7th International African Palliative Care Conference, held from the 24th to 26th August 2022 in Kampala, Uganda and virtually

**DOI:** 10.3332/ecancer.2022.1474

**Published:** 2022-11-23

**Authors:** Julia Downing, Eve Namisango, Stephen Connor, Patricia Batanda, Lisa Christine Irumba, Berna Basemera, Alfred Jatho, Sylvia Nakami, Harriet Nalubega, Antonia Kamate, Dianah Basirika, Joyce Zalwango, Mable Namuddu, Wedzerai Chiyoka, Francis Kayondo, David Byaruhanga, Eugene Rusanganwa, Helena Davis, Stephen Watiti, Babe Gaolebale, Lacey N Ahern, Lydia Thomas, Emmanuel Luyirika

**Affiliations:** 1 Makerere/Mulago Palliative Care Unit, Kampala, Uganda; 2 International Children’s Palliative Care Network, Durban 3624, South Africa; 3 African Palliative Care Association UK, London DA7 6AZ, UK; 4 African Palliative Care Association, Kampala, Uganda; 5 Worldwide Hospice Palliative Care Alliance, London WC1X 9JG, UK; 6 Palliative Care Association of Uganda, Kampala, Uganda; 7 Uganda Cancer Institute, Kampala, Uganda; 8 Rays of Hope Hospice Jinja, Jinja, Uganda; 9 Hospice Africa Uganda, Kampala, Uganda; 10 Eck Institute for Global Health, University of Notre Dame, Notre Dame, IN 46556, USA; 11 Hospice Foundation/Global Partners in Care, Mishawaka, IN 46545, USA

**Keywords:** cancer care, palliative care, Africa, COVID-19, pandemic, policy, integration, education, research, paediatrics, humanitarian, digital

## Abstract

The 7th International African Palliative Care Conference and the 4th African Ministers of Health Meeting were held in Kampala from the 24th to 26th August 2022. The theme of the conference – *Palliative Care in a Pandemic* – reflected the reality of palliative care provision on the continent, and the experience of patients and providers over the past 2 years. It was hosted by the African Palliative Care Association and the Worldwide Hospice Palliative Care Alliance with co-sponsors being the International Children’s Palliative Care Network, the International Association of Hospice and Palliative Care, Global Partners in Care and Palliative care in Humanitarian Aid Situations and Emergencies. The conference was held in Kampala as a hybrid event, with a mix of in-person, pre-recorded and virtual presentations. The African Ministers of Health Meeting held on the 24^th^ August was attended by delegates from 25 Ministries of Health, with 92 participants in-person and 122 attending virtually. Hosted by the Minister of State for Primary Health Care in Uganda, the participants at the meeting endorsed a *Declaration on Palliative Care in a Pandemic*. The main conference, held on the 25th and 26th August, was attended by 334 delegates from 40 countries, 199 (60%) of whom attended in-person. Key themes discussed throughout the conference included: contagious compassion; building a business case and evidence for palliative care in Africa; palliative care policy, funding and sustainability; the importance of collaboration and global partnerships; palliative care for all ages, children through to the elderly, and all conditions; the need to be innovative and creative, embracing technology; and a feeling of hopefulness in the future of palliative care in the region as we go forward together. The impact of the pandemic has been significant on everyone. Despite this, and the limitations imposed by the pandemic, the African palliative care community has come through it stronger, is committed to continuing the development of palliative care across the region, working together and is hopeful for the future.

## Introduction

In countries all around the world, the pandemic has firmly placed the spotlight on mortality. COVID-19 has taken lives in every country and has resulted in people talking about death, dying and bereavement [1]. Across Africa, the pandemic has resulted in lockdowns, in harsh economic climates and in health systems and health professionals struggling to cope with the impact of the pandemic. However, despite this, the African Palliative Care Association (APCA) has sought to bring individuals and organisations together from across the region virtually, with this 7th International African Palliative Care Conference finally bringing people together in person as well as on-line, through the hybrid nature of the conference. The theme of the conference, *‘Palliative Care in a Pandemic’* reflects the reality of palliative care provision on the continent, the experience of patients and providers over the past 2 years and the projected risks of emerging infectious diseases in Africa in the future. From the first APCA conference held in Tanzania in 2004, conferences have been held in Kenya (2007), Namibia (2010), South Africa (2013), Uganda (2016) and Rwanda (2019) each providing opportunities for palliative care providers to exchange knowledge, share lessons learnt, disseminate research findings and learn from each other. This 7th conference provided a forum for setting new palliative and comprehensive chronic care implementation agendas based on the new global frameworks and emerging global security issues. Palliative care providers and supporters were able to review progress on the African continent and advocate for more palliative and comprehensive chronic care development.

Pre-conference workshops were held on the 22nd and 23rd August addressing issues around children’s palliative care, research and digital health in palliative care. These were followed on the 24th August by the 4th African Ministers of Health Meeting, with the main conference held on the 25th and 26th August 2022 at the Mestil Hotel, Kampala. The conference was hosted by APCA and the Worldwide Hospice Palliative Care Alliance (WHPCA). APCA is a membership non-profit pan-African organisation committed to reducing pain and suffering for people living with life-limiting and life-threatening conditions across Africa. APCA was established in 2002 with its head office in Kampala, Uganda. APCA’s vision is access to palliative and comprehensive care for all in Africa, with a mission to ensure palliative and comprehensive chronic care is understood and integrated into health systems at all levels to reduce pain and suffering across Africa [2, 3].

The WHPCA was established in 2008 as a global network to be a strong voice advocating for hospice and palliative care in the international arena. WHPCA is now an international alliance of 400 national and regional hospice and palliative care and affiliate organisations representing 103 countries. WHPCA’s vision is a world with universal access to quality palliative care, with a mission to bring together the global palliative care community to improve well-being and reduce unnecessary suffering for those in need of palliative care in collaboration with the regional and national hospice and palliative care organisations and other partners [4]. The co-sponsors of the conference were the International Children’s Palliative Care Network (ICPCN), the International Association of Hospice and Palliative Care (IAHPC), Global Partners in Care and Palliative care in Humanitarian Aid Situations and Emergencies (PallCHASE).

Access to health services, including palliation, ensures healthier people and is a key element to reducing social inequities. Whilst progress has been made in building strong global frameworks to enhance the provision and access to palliative and comprehensive chronic care and other essential health services, including essential medicines, the pandemic has impacted greatly on service delivery. Palliative care is recognised as an essential service under Universal Health Coverage (UHC) [5] and as part of Primary Health Care through the Declaration of Astana [6]. The World Health Organization (WHO) states that all people should have access to the health services they need, including palliative care, without the risk of financial hardship when paying for them [5]. This was reiterated during the pandemic in the UHC2030 Report on State of Commitments to UHC which stated that ‘*No one should face financial, geographical or cultural barriers to access to essential COVID-19- related services including testing, treatment, palliative care and vaccines, once they become available’* [7]. This builds on both the World Health Assembly (WHA) resolutions on palliative care in 2014 [8] and cancer care in 2017 [9], along with the Lancet Commission report on alleviating the access abyss to pain and palliative care [10].

Morbidity across the region remains high, with Africa having over 75% of all HIV cases globally [11], with late-stage presentation and inaccessible diagnosis and treatment being common [12] and with many countries still lacking access to effective pain control and appropriate treatment such as radiotherapy [13]. Africa has the greatest need for palliative care per population, with HIV/AIDS dominating over malignant and other non-malignant diseases. In children, Africa accounts for over half the need for palliative care, which is mostly due to HIV. Cancer accounts for a smaller percentage of overall need in children, with premature birth, birth trauma and congenital malformations together accounting for the highest palliative care need in children [14]. Within the region, co-morbidities such as HIV and tuberculosis, along with advanced cancer, posed a higher risk of mortality for individuals with COVID-19. Alongside this, the care of individuals with COVID-19 must include palliative care, with palliative care included in case management guidelines [15].

## The 4th African Ministers of Health Meeting

The meeting was held on Wednesday 24th August, with participants attending both in person (92) and virtually (122). Delegations from 25 African Ministries of Health attended including Uganda, Benin, Botswana, Burkina Faso, Cameroon, the Democratic Republic of Congo (DRC), Egypt, Ethiopia, Gabon, Ghana, Kenya, Lesotho, Liberia, Malawi, Morocco, Mozambique, Namibia, Nigeria, Rwanda, Senegal, South Africa, Sudan, Tanzania, Zambia and Zimbabwe. Participants were welcomed by Dr Emmanuel Luyirika, the Executive Director of APCA, who set the scene for the purpose of the meeting and introduced the national delegations and palliative care development partners. The Hon. Margaret Muhanga Mugisa, Minister of State for Primary Health Care in Uganda, opened the meeting, welcoming participants to Uganda and the Minister’s meeting. She highlighted the importance of working together as Ministries of Health across the region to improve the provision of palliative care services. Presentations covered a range of issues including: the state of UHC and the inclusion of palliative care and other essential services, Dr Yonas Tegegn Woldemariam, WHO representative in Uganda; investment in radiotherapy to support palliative care, Dr Lisa Stevens, Director, International Atomic Energy Agency-Programme of Action for Cancer Therapy (IAEA-PACT); and palliative care in a pandemic, Dr Patrick Kagurusi, Amref Health Africa. Dr Tedros Adhanom Ghebreyesus, the Director General of the WHO made a recorded presentation on the topic and then we heard from some family members of palliative care beneficiaries and then a discussion was held on the role of regional bodies in achieving UHC which was contributed to by Africa Centres for Disease Control and Prevention (CDC), Amref and IAEA-PACT. The key points included engaging community-based health workers, adopting online platforms to take services closer to beneficiaries and governments taking advantage of existing funding mechanisms to increase access to radiation technology for diagnosis, treatment and palliation for cancer and other conditions.

In the afternoon, several ministries presented on palliative care development and its integration in national health systems in their respective countries, focusing on policy, human resources, medicines and technologies, service delivery, financing, data collection and plans for palliative care as part of UHC. It was clear from the presentations that countries have reached different levels of palliative care integration, training, research and policy formulations. It was also evident that countries see education and training as essential in order to increase the human resources for palliative care. Some of the countries such as Namibia and Botswana highlighted the need to increase the numbers of health workers with appropriate palliative care skills through training and education. This was followed by a panel discussion which was moderated by Professor Rhoda Wanyenze the principal, School of Public Health, Makerere University, who engaged the following panellists: Lisa Stephens from IAEA; Abdulaziz Mohammed – Africa CDC; Lamia Mahmoud, WHO Regional Office for the Eastern Mediterranean; and Dr Patrick Kagurusi, Amref. The panellists discussed: existing opportunities which African countries can leverage on to achieve UHC; how palliative care services can reposition themselves to build resilient health systems after the COVID-19; and how palliative care services can reposition to benefit from emerging funding streams amidst the current global health challenges in order to build resilient systems which are inclusive of UHC.

**Figure 1.**
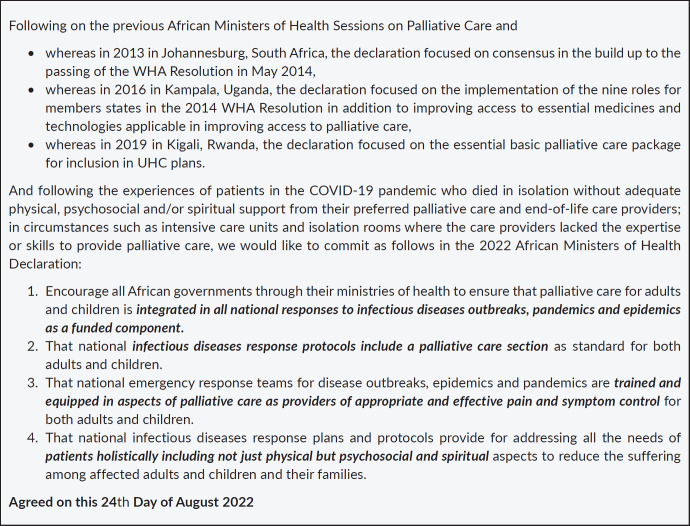
The Declaration on Palliative Care in a Pandemic.

It was also acknowledged that funding for palliative care is in many instances outside of the government systems with the majority of services provided by the non-governmental sector and much funding has been cut across countries. However, political leaders have the will and through their ministries of health are supportive of palliative care development in their countries. Cross-cutting challenges to palliative care development included limited access to essential medicines, the low priority attached to palliative care in comparison to other health services and that there are funding gaps for palliative care service delivery, research and education. At the end of the meeting, those present signed a Declaration on Palliative Care in a Pandemic (Figure 1).

## Opening of the conference

In the opening plenary on the 25th August, following a thanksgiving by Father Rick Bauer, Dr Emmanuel Luyirika (APCA) and Dr Stephen Connor (WHPCA) welcomed and thanked the conference delegates from across the world attending and making the conference possible, stressing that we need to continue advocating for palliative care to be universally accessible across the region. They then welcomed two Ugandan cancer patients to the stage who shared their experiences and gave testimony to the impact of palliative care on their lives and those of their families. Two other patients from Zimbabwe also shared their testimonies via film on how care provided through the community-based palliative care programme had given them new hope and improved their quality of life.

The keynote address on ‘Palliative care in pandemics’ was given by Professor Liz Gwyther from South Africa. Speaking virtually, she noted the importance of palliative care being integrated throughout the pandemic response, ensuring the service is available not just for pre-existing palliative care patients, but also for those in need through the pandemic. She shared some lessons learnt from South Africa and the need to strengthen the public health response and policy when providing palliative care during a pandemic. Hon. Margaret Muhanga Mugisa, Minister of State for Health in Charge of Primary Health Care, then officially opened the conference, welcoming participants and encouraging them to continue to develop services and train more professionals in palliative care in order to meet the ever-increasing needs for palliative care services across the region.

## Conference summary

The conference, held in-person at the Mestil Hotel, Kampala, and virtually, brought together 334 delegates – 199 (60%) of whom attended in person and the rest (135 – 40%) virtually. This was the first time that the conference had been held in a hybrid format and was in recognition of the ongoing travel challenges due to COVID-19 restrictions and requirements and the economic impact of the pandemic resulting in many delegates being unable to travel and attend in person. Delegates attended from a range of African countries including: Benin, Botswana, Burkina Faso, Cameroon, DRC, Eswatini, Ethiopia, Gabon, Ghana, Kenya, Lesotho, Malawi, Morocco, Mozambique, Namibia, Nigeria, Rwanda, Senegal, Seychelles, Sierra Leone, South Africa, South Sudan, Sudan, Tanzania, The Gambia, Uganda, Zambia and Zimbabwe. As anticipated, Uganda had the most participants attending in-person. Delegates attended from around the world including Australia, Canada, the Czech Republic, Malaysia, Nepal, Peru, Portugal, Spain, Taiwan, Singapore, the United Kingdom and the United States of America. This brought together a range of palliative care providers, patient groups, academics, researchers, donors, development partners, commissioners and policymakers, advocates and government representatives. Networking was key, and there were opportunities to network both online and in-person. There were three plenary sessions with 16 presentations, a panel discussion, five workshops and 100 concurrent oral presentations across five conference tracks. The conference was structured around five tracks, and each track included cross-cutting issues such as inter-disciplinary teams, paediatric palliative care, communicable and non-communicable diseases, mental health, ageing, spiritual care and utilising disease prevention opportunities in the process of designing and implementing palliative care. The five tracks were:

1. Empowerment through education/learning
2. Building an African evidence-base
3. Comprehensive/holistic care
4. Harnessing technology in palliative care
5. Health systems, policy and law.

Alongside the oral presentations, there were also 72 poster presentations, some of which were virtual, and some physical. Thus, there were many opportunities to learn from each other and to hear what is happening across the region. All sessions during the main conference were live-streamed, with up to five tracks being streamed concurrently with presenters online and in the room. Despite this complexity, the conference went well, with only minor technical challenges being addressed as they arose.

## Key conference themes

A wide range of issues were discussed throughout the conference. Some key themes identified included:

1. Contagious compassion
2. Building a business case and evidence for palliative care in Africa
3. Palliative care policy, funding and sustainability
4. The importance of collaboration and global partnerships
5. Palliative care for all ages, children through to the elderly and all conditions
6. The need to be innovative and creative, embracing technology
7. A feeling of hopefulness in the future of palliative care in the region as we go forward together.

### Contagious compassion

From the start of the conference with the opening words from APCA, WHPCA, the Minister of Health, The WHO, patients and their families and the keynote address, the importance of compassion was highlighted. Compassion is not only the awareness and empathy of suffering but the commitment to take action to relieve the suffering. Compassion is inherently personal and has been seen as ‘providing the un-spoken language to address unspeakable suffering’ [16] (p937). Larkin suggests that *‘Compassion requires resilience, fortitude and sometimes risk-taking, but always tenacity and determination’* [17] (p7) and is core to the provision of palliative care. Throughout the pandemic, and throughout the development of palliative care across the region, there is a feeling of ‘Contagious Compassion’ – where compassion spreads to and affects others – where the compassion shown in the provision of palliative care is seen, recognised and spreads to all it touches. So important throughout the pandemic, and as we demonstrate the need for palliative care, the outcomes of the care that we provide, as we address education and training, and as well ensure that palliative care is recognised within the pandemic response, compassion is at the root of all that we do. Throughout the tracks and the plenary presentations, compassion was demonstrated through stories, research, outcomes of care, the driver for advocacy and seen both physically and digitally.

### Building a business case and evidence for palliative care across Africa

One of the plenary presentations on day 1 was on *‘Building a business case for palliative care within Universal Health Coverage for low- and middle-income countries: What is the evidence?’* Dr Jane Bates, formally at Kamuzu University of Health Sciences in Blantyre, Malawi, shared the results of her PhD study which aimed to make a business case for palliative care. Whilst we have been good at making an emotional case for palliative care, this has not always been backed up by a sound business case for palliative care. We need to convince our governments, our donors and our organisations to invest in palliative care. She shared ten principles of building a business case for palliative care within UHC in Africa. These were first outlined in a paper by Cassel *et al* [18] on the development of a business case for palliative care in the US. These can be adapted and utilised within the African context to bring about the changes needed, many of which were highlighted in the Lancet commission report in 2017 [10]. Dr Bates emphasised that the evidence base to support a business case for palliative care relevant to African countries is growing and we can continue to develop it further.

Throughout the conference, speakers were presenting some of the evidence that will help build the business case, and it was encouraging to see the range of research studies presented both during the pre-conference workshop and throughout the conference from across the region – the African evidence base is growing and we need to utilise it to build our business case for palliative care.

### Palliative care policy, funding and sustainability

**Figure 2.**
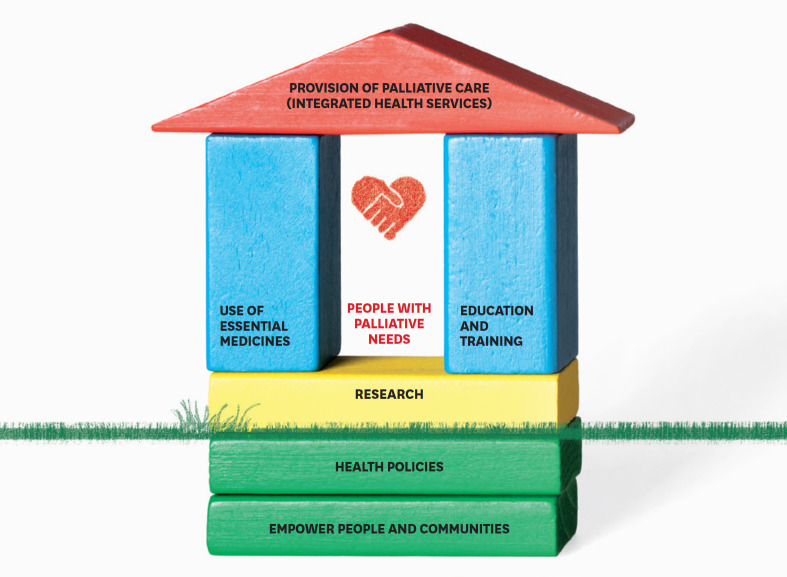
Conceptual model for palliative care development [19] (p14).

Policy for palliative care is essential, and throughout the conference the need for policy was discussed – policy for palliative care at the national, regional and international levels, but also the importance of palliative care being included in policies related to the pandemic. In 2021, the WHO conceptual model was published alongside indicators for palliative care [19] (Figure 2). The conceptual model builds upon the previous foundation measures for palliative care published initially in 2006 [20] which highlighted the need for policy, drug availability, education and implementation, with the addition of research as the fifth pillar in 2013 [21]. Health Policy is key and refers to ‘*the political commitment and leadership expressed in governance and policy frameworks. It includes the development of a legal framework and regulations that guarantee the rights of patients, access to palliative care services and essential medicines, and the financing and inclusion of palliative care in the National Health Service and benefits package. It also includes health system design and health care organisation, in addition to stewardship and multi-stakeholder action’* [19] (p15).

Throughout the conference, issues of policy were highlighted, both in terms of the importance of having a policy, the challenges in getting policies approved and the impact of policies in terms of financing and sustainability. Throughout the tracks, issues of policy, funding and sustainability were highlighted as challenges, with limited examples of how these challenges can be overcome. Funding for palliative care across the region was reducing prior to the pandemic, and the impact of the pandemic on funding has been catastrophic. Whilst during the acute phase of the pandemic, funding for COVID-19 was available, this has now reduced, and funding for ongoing palliative care service provision is lacking. Sustainability of organisations is essential, but how we manage that – and how we ensure that services are integrated and that governments funding care remains challenging.

### The importance of collaboration and global partnerships

*‘If you want to go quickly, go alone. If you want to go far, go together’*

(African Proverb)

Never has this African proverb been more true. We need to go together to go far, to continue to develop palliative care across the region, to learn from each other, to support each other and to encourage each other. Whilst recognising that limited funding options can cause competition, it was also recognised that if we don’t collaborate, if we don’t work together, then we will not be able to continue to develop and strengthen palliative care within the region. However, not only do we need to collaborate with each other in-country and within the region, but we need global partnerships. Such partnerships and collaborations were emphasised across all of the conference tracks. Through collaboration and partnerships, we can strengthen education and training opportunities, supporting, mentoring and empowering others. Much of the research being done across the region is in collaboration between organisations in the region along with global research partners such as Kings College London, the University of Edinburgh, St. Jude’s Global and the University of Notre Dame. In our efforts to advocate with governments, with regional and global organisations, we are stronger as we go forward together, working hand in hand from different organisations, countries, backgrounds (e.g. paediatric, adult, the elderly) we will go further.

### Palliative care for all ages, children through to the elderly, and all conditions

Cross-cutting themes throughout the conference included children’s palliative care, care of the elderly, care in humanitarian settings, communicable and non-communicable disease and mental health. Plenary sessions highlighted the need for mental health in palliative care, palliative care within sickle-cell disease, the essential package for cervical cancer, children’s palliative care, caring for the caregiver and learning from advocacy in other fields of health and human rights. The WHO definition of palliative care [22] encompasses caring for all individuals with life-limiting and life-threatening conditions, regardless of the type of condition, their age, where they are being cared for thus these cross-cutting themes were essential as we focused on palliative care in a pandemic. A range of models of palliative care delivery were discussed and the APCA Network for Primary Palliative Care was launched during the first day of the conference. Alongside this, a pre-conference workshop was held on palliative and end-of-life care in paediatrics by APCA, ICPCN and St. Jude’s Global. The workshop was split into two groups – those learning about children’s palliative care for the first time and those with advanced skills and seen as leaders within the field.

### The need to be innovative and creative, embracing technology

Technology is here with us to stay. Prior to the pandemic, many had been reluctant to embrace technology due to challenges in bandwidth, access to the Internet or access to equipment. Yet this has changed through the pandemic. Presentations shared the development of e-learning programmes, online training, webinars, strengthening the supply chain through m-health, remote case presentations and consultations. Whilst challenging, and whilst we may not have been as prepared as we could have been [15], we have embraced technology. The pre-conference workshop on digital health in palliative care showcased recent projects involving the development and evaluation of digital health approaches for palliative care in sub-Saharan Africa. It explored the practical challenges of undertaking digital health research alongside how factors of sustainability and scale-up could be explored. Throughout the conference, presenters shared ways in which they had been innovative and creative in the care that they provided throughout the pandemic, much of which will be continued and developed as we move forward together.

### A feeling of hopefulness in the future of palliative care in the region as we go forward together

It was acknowledged that the past few years have been tough for everyone – for our patients and families and for each of us working in palliative care. Many of us have lost loved ones, have seen things that we would rather not have seen and have had to think about different ways of doing things. Yet despite this, there was a feeling of hopefulness as we move forward. Importantly, it was recognised that we had all ‘done what we could’ during the challenging times – we had risen to the occasion, and whilst not ideal, we had done our best. Yes, it has been tough; yes, we have had challenges, but we are still here and still delivering compassionate care to those in need. We have been resilient, we have been innovative and creative, we have embraced a ‘new normal’ and we have moved forward and learnt together. As we begin to come out of the pandemic, we are moving forward together, we are stronger because of what we have been through, we can build on the broader understanding of the need for palliative care that the pandemic has brought, and we can increase accessibility to quality palliative care services for all in need across the region.

## Conclusion

The impact of the pandemic has been significant on everyone. Despite this, and the limitations imposed by the pandemic, we have come through it stronger, we are committed to continuing the development of palliative care across the region, we are working together and we are hopeful for the future.

As the conference came to a close, Dr Eve Namisango, Chair of the Scientific Committee, Dr Emmanuel Luyirika, Executive Director of APCA, and Professor Julia Downing, Vice-Chair of the Scientific Committee, thanked all those involved. Professor Downing summarised the conference, highlighting key points and sharing a few thoughts. Dr Namisango announced the awards for the best oral abstracts (Integrating palliative care data into the National Health Management Information System during the COVID-19 pandemic – *Lead Author: Cynthia Kabagambe, Palliative Care Association of Uganda;* Pain management practices among nurses providing end-of-life care at the Komfo Anokye Teaching Hospital (KATH), Ghana – *Lead Author: Lydia Asamoah, KATH, Ghana;* and Incorporation of legal aspects in palliative care – *Lead Author: David Musyoki, Kenya Hospices and Palliative Care Association (KEHPCA), Kenya*) and the best poster abstracts (Evaluating the use of WhatsApp during the COVID-19 pandemic for remote multi-disciplinary meetings and consultations of a palliative care team in northeastern Congo – *Lead Author: Patricia Strubbe Aru Palliative Care Team, DRC;* Review of APCA Standards to Malawi standards – *Lead Author: Glenda Winga Palliative Care Association of Malawi*)*.* Dr Luyirika also thanked the Hon. Minister from Namibia who had attended the whole of the conference in person, and is looking forward to further collaboration with the Ministry in Namibia. Dr Stephen Connor thanked all global partners and Dr Justin Baker concluded the meeting with prayerful reflections and thanksgiving on the activities of the week.

All agreed that it had been an exciting conference to be part of and are already looking forward to the next one. After the conference, Professor Anne Merriman (Founder of Hospice Africa Uganda) summed it up by saying: ‘*This was the most wonderful APCA conference that I have attended. It was particularly great to be here because I met people from the many countries in Africa where we have introduced palliative care…. The plenary speakers were good. The amount of knowledge in that conference captured both with the speakers and the posters which came from many countries in Africa is phenomenal’.*

## Conflicts of interest

The authors declare that they have no conflicts of interest.
